# A Novel Dehumidification Strategy to Reduce Liquid Fraction and Condensation Loss in Steam Turbines

**DOI:** 10.3390/e23091225

**Published:** 2021-09-18

**Authors:** Yan Yang, Haoping Peng, Chuang Wen

**Affiliations:** 1 School of Petroleum Engineering, Changzhou University, Changzhou 213164, China; yyan-petroleum@cczu.edu.cn (Y.Y.); penghp@cczu.edu.cn (H.P.); 2 College of Engineering, Mathematics and Physical Sciences, University of Exeter, Exeter EX4 4QF, UK

**Keywords:** wet steam, two-phase flow, phase change, steam turbine, blade cascade, dehumidification

## Abstract

Massive droplets can be generated to form two-phase flow in steam turbines, leading to erosion issues to the blades and reduces the reliability of the components. A condensing two-phase flow model was developed to assess the flow structure and loss considering the nonequilibrium condensation phenomenon due to the high expansion behaviour in the transonic flow in linear blade cascades. A novel dehumidification strategy was proposed by introducing turbulent disturbances on the suction side. The results show that the Wilson point of the nonequilibrium condensation process was delayed by increasing the inlet superheated level at the entrance of the blade cascade. With an increase in the inlet superheated level of 25 K, the liquid fraction and condensation loss significantly reduced by 79% and 73%, respectively. The newly designed turbine blades not only remarkably kept the liquid phase region away from the blade walls but also significantly reduced 28.1% averaged liquid fraction and 47.5% condensation loss compared to the original geometry. The results provide an insight to understand the formation and evaporation of the condensed droplets inside steam turbines.

## 1. Introduction

A steam turbine is one of essential components to achieve energy conversion and utilisation for industrial applications [1,2,3,4,5]. The condensation process occurs due to the steam expansion in the low-pressure stage and subsequently forms two-phase flows in steam turbines [6,7,8]. The experimental studies have also demonstrated the distribution of the polydispersed droplets in blade cascades [9,10,11]. The high-speed wet steam flow containing massive droplets not only results in the erosion issue but also affects the reliability of the machinery [12,13,14]. Therefore, it is crucial to improve the dehumidification for steam turbines. The dehumidification characteristics were achieved by optimising the structures [15] and operating conditions [16], which were based on the nonequilibrium condensation in transonic flows.

Numerical approaches have been employed to model the condensing two-phase flows in steam turbines [17,18,19]. The polydispersed droplet distribution was studied using numerical modelling in blade cascades in steam turbines. White and Hounslow [20] used the moment method to describe the distribution of the droplet sizes in the condensing flow in steam turbines and they found that the moment method showed good agreement with experimental data while reducing the computation cost. White [21] also compared the mixed Eulerian–Lagrangian, fully Eulerian and moment methods in predicting the distribution of the droplet sizes in wet steam flows. Gerber and Mousavi [22] investigated the accuracy and robustness of the quadrature method of moment in modelling the distribution of the droplet sizes in the two-phase flow in low-pressure turbines. Furst et al. [23] developed a pressure-based solver to model the wet steam flow in OpenFOAM and successfully predicted the nonequilibrium condensation in turbine blades. Aliabadi et al. [24] proposed a new method by injecting the hot steam into the blade cascades and evaluated the effect of the locations of the injections on the wet steam flows. Hoseinzade et al. [25] studied the effect of volumetric heating rates on the steam flow and found the optimised values to reduce the wet steam condensations in steam turbines. Han et al. [26] developed a numerical model to evaluate the effect of surface heating on the condensation behaviour in steam turbines and subsequently optimised the blade performance. Post et al. [27] compared the Reynolds-averaged Navier-Stokes (RANS) and large eddy simulation (LES) models for the condensing flow in turbine blades, which showed that the LES method was better in modelling the transient condensing flow and shock waves. The aforementioned numerical studies performed an understanding of the unsteady nucleation and condensation processes in the blade cascades in steam turbines.

The purpose of this study was to develop a condensing two-phase flow model to predict the transient nucleation and condensation processes because of high expansion characteristics in steam turbines. A novel dehumidification strategy was proposed via generating turbulent disturbances in the flow field, which can be achieved by installing a delta wing on the blade wall on the suction side. The condensation mechanism and losses were analysed within the newly designed turbine blades to show the advances of the novel dehumidification strategy.

This study is organised as follows: the numerical implementation and geometrical model are briefly described in Section 2, including the governing equations, numerical methods and blade geometries for the steam turbine. Results and discussion are shown in Section 3, including the model validations for the wet steam flows in the converging–diverging nozzle and steam turbines, the influence of the superheated steam and a new strategy in reducing the liquid fractions in blade cascades. The conclusion is summarised in Section 4.

## 2. Numerical Model

In the present study, the two-dimensional (2D) simulation is carried out based on the wet steam two-phase flow model [28,29]. The phase change is determined by the nonequilibrium condensation behaviour due to the high expansion process of the steam in the transonic flows [30,31]. The conservation equations that govern the wet steam flow in the blade cascades can be written as:

(1) $$\frac{\partial (H)}{\partial t}+\frac{\partial \left(U_{x}\right)}{\partial x}+\frac{\partial \left(U_{y}\right)}{\partial y}=\frac{\partial \left(J_{x}\right)}{\partial x}+\frac{\partial \left(J_{y}\right)}{\partial y}+S$$

where:

(2) $$H=\left[\begin{array}{l} \rho \\ \rho u\\ \rho v\\ \rho E\\ \rho n\\ \rho y \end{array}\right],U_{x}=\left[\begin{array}{l} \rho u\\ \rho uu+p\\ \rho uv\\ \rho u(E+p)\\ \rho un\\ \rho uy \end{array}\right],U_{y}=\left[\begin{array}{l} \rho v\\ \rho uv\\ \rho vv+p\\ \rho v(E+p)\\ \rho vn\\ \rho vy \end{array}\right]$$

(3) $$J_{x}=\left[\begin{array}{l} 0\\ \tau_{xx}\\ \tau_{xy}\\ q_{x}\\ 0\\ 0 \end{array}\right],J_{y}=\left[\begin{array}{l} 0\\ \tau_{xy}\\ \tau_{yy}\\ q_{y}\\ 0\\ 0 \end{array}\right],S=\left[\begin{array}{l} -\dot{m}\\ -u\dot{m}\\ -v\dot{m}\\ -(h_{v}-h_{fg})\dot{m}\\ \rho I\\ \dot{m} \end{array}\right]$$

where *H*, *U* and *J* represent the conservation variables, inviscid and viscid fluxes. *n* and *y* describe the liquid number and fraction. $\dot{m}$ is the condensation mass rate due to the phase change of steam in transonic flows [32,33]:

(4) $$\dot{m}=\frac{4\pi r_{c}{}^{3}}{3}\rho_{l}I+4\pi r^{2}\rho_{l}n\frac{dr}{dt}$$

(5) $$I=\frac{q_{c}}{1+\phi }\frac{\rho_{v}{}^{2}}{\rho_{l}}\sqrt{\frac{2\sigma }{\pi m_{v}{}^{3}}}\exp\left(-\frac{4\pi \sigma }{3k_{B}T_{v}}r_{c}{}^{2}\right)$$

(6) $$\frac{dr}{dt}=\frac{\lambda_{v}\left(T_{s}-T_{v}\right)}{\rho_{l}h_{lv}r}\frac{\left(1-r_{c}/r\right)}{\left(\frac{1}{1+2\beta \mathrm{Kn}}+3.78(1-\nu )\frac{\mathrm{Kn}}{\mathrm{Pr}}\right)}$$

where *I* and *dr*/*dt* are the nucleation rate and droplet growth rate [34,35].

The shear stress transport (SST) *k*-*ω* turbulence model [36,37,38] is suggested for the prediction of the condensation phenomenon in transonic flows.

(7) $$\frac{\partial }{\partial t}(\rho k)+\frac{\partial }{\partial x_{i}}(\rho ku_{i})=\frac{\partial }{\partial x_{j}}\left(\Gamma_{k}\frac{\partial k}{\partial x_{j}}\right)+{\bar{G}}_{k}-Y_{k}+S_{k}$$

(8) $$\frac{\partial }{\partial t}(\rho \omega )\frac{\partial }{\partial x_{j}}(\rho \omega u_{j})=\frac{\partial }{\partial x_{j}}\left(\Gamma_{\omega }\frac{\partial \omega }{\partial x_{j}}\right)+C_{\omega }-Y_{\omega }+D_{\omega }+S_{\omega }$$

where *Γ_k_* and *Γ_ω_* are the effective diffusivity of *k* and *ω*, respectively.

The numerical simulation is carried out based on the commercial platform ANSYS Fluent 18.2. The mass, momentum and energy equations are directly solved by ANSYS Fluent, while the phase change process was integrated into Fluent using User-Defined Scalar (UDS) Transport Equations and User-Defined Functions (UDF) using C code. The droplet number and liquid fraction are solved by two additional scalar equations by UDS, while the source terms are employed to describe the mass generation during nonequilibrium condensations in transonic flows by UDF. The governing equations are discretised by the finite volume method, and the density-based solver is employed for wet steam flows. The second-order upwind discretisation is used for the flow, turbulence and droplet number and liquid fraction equations. The initialisation for the numerical simulation is performed by computing the inlet parameters of the computational cases. The pressure inlet and pressure out boundaries are assigned to the entrance and exit of the blade cascades in the steam turbine. The convergence criteria are below 1.0 × 10^−4^ for all dependent variables and the relative difference of mass flow rate between the inlet and out boundaries is lower than 0.0005%.

**Figure 1:**
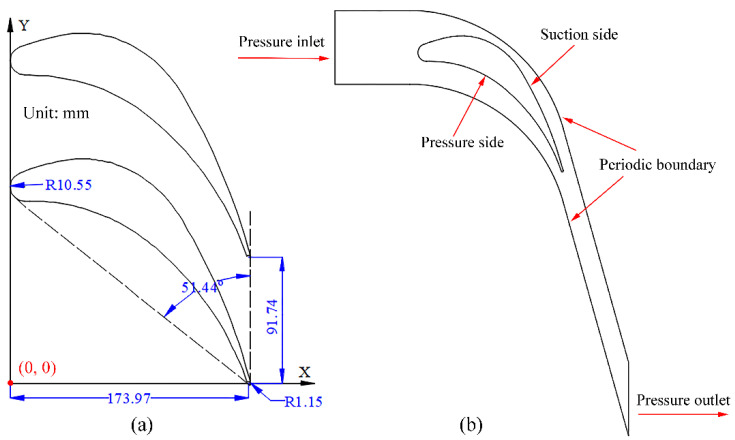
Physical model of the blade cascade in steam turbines: (**a**) geometric model and (**b**) boundary conditions.

**Figure 2:**
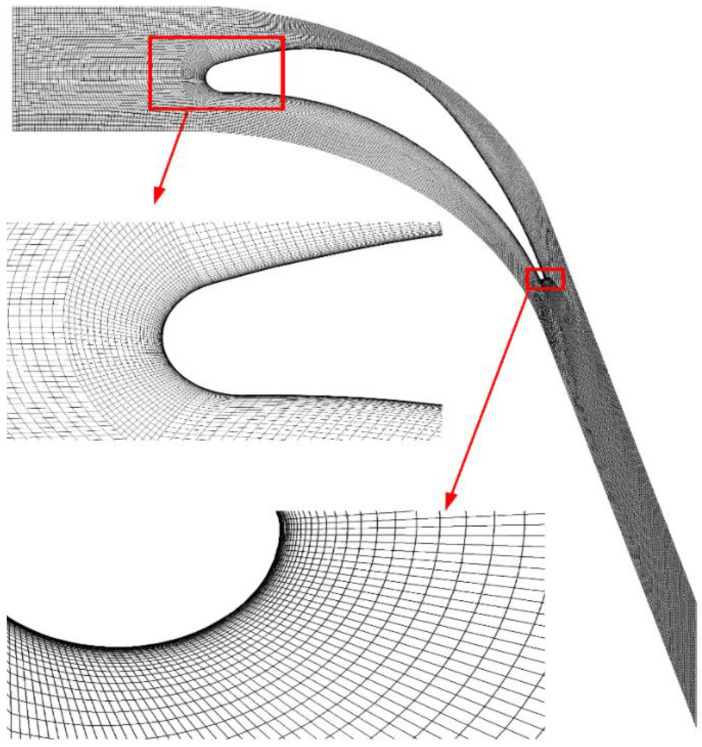
Computational grid of the blade cascade.

The geometry and operating parameters are borrowed from Dykas et al. [39] linear blade cascade in a low-pressure steam turbine. Figure 1 shows the geometrical dimensions and boundary conditions that are performed in the numerical implementation. Table 1 and Table 2 list the detailed dimensions and operating conditions for the linear blade. The numerical simulation is carried out based on the structured grid system, as shown in Figure 2, while three different numbers of 60,076, 120,552 and 240,405 elements are employed for the mesh independence test.

**Figure 3:**
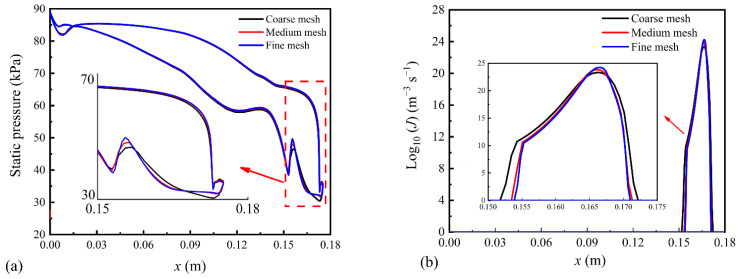
Effect of grid densities on flow parameters in the steam turbine: (**a**) static pressure and (**b**) nucleation rate.

Figure 3 describes the distributions of the wall pressure and nucleation rate in the blade cascade based on various grid densities. It can be seen that three grid systems predict almost the same wall pressures while the coarse grid system induces an earlier Wilson point of nucleation processes compared to other two cases. Additionally, the coarse mesh calculates a larger nucleation region than medium and fine grid systems. Hence, the medium mesh of 120,552 cells is employed for the prediction of the two-phase flow in the steam turbine.

## 3. Results and Discussion

### 3.1. Wet Steam Model Validation in Transonic Flows 

#### 3.1.1. Wet Steam Flow in Converging-Diverging Nozzles

**Figure 4:**
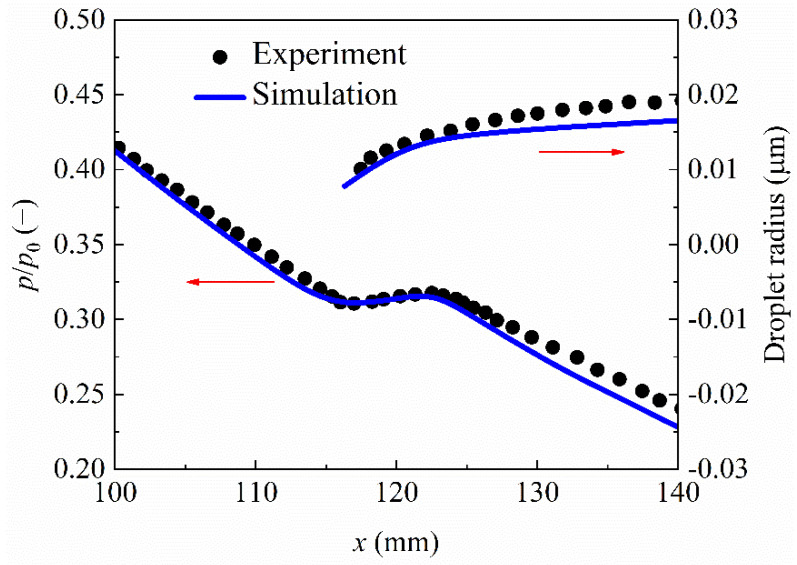
Static pressure and droplet size in the converging-diverging nozzle [36].

The case 203 in Moses and Stein’s converging-diverging nozzle [40] is used for the validation of the steam condensation in supersonic flows. The detailed geometry and operating parameters can be found in the published paper [40]. The comparison between the numerical and experimental values are depicted in Figure 4, including the static pressure and droplet radius along the flow direction. We can see that both the numerical pressures and droplet sizes agree well with experimental measurements although the predicted values are lightly smaller than the experimental data in the downstream region of condensing shocks. This indicates that the developed condensing flow model can capture Wilson point of nucleation processes and predict the generation of the condensed liquid phase. 

#### 3.1.2. Wet Steam Flow in Steam Turbine Blades

**Figure 5:**
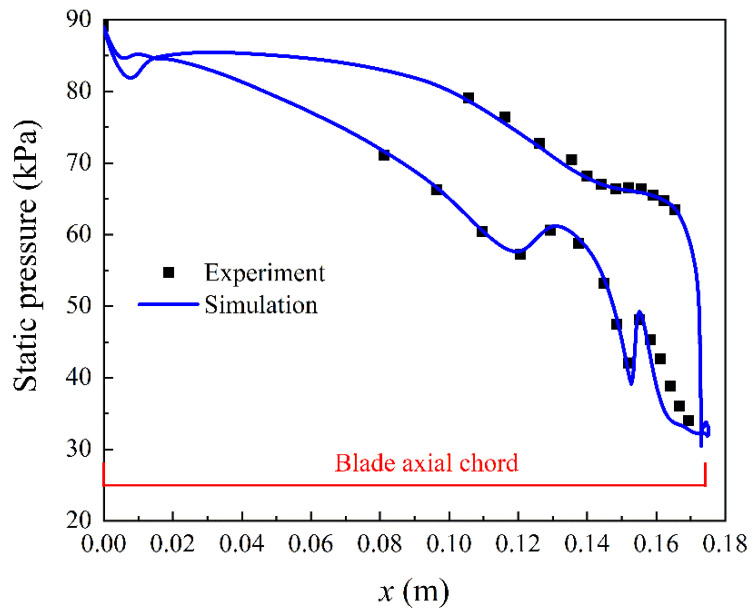
Numerical and experimental wall pressure of a steam blade [39].

The developed condensing flow model is also validated in a steam turbine [39]. The computed wall pressures are compared with the experimental values in Figure 5. It can be observed that the numerical wall pressures are good with experimental measurements on both the suction side and pressure side. The condensation shock due to the nonequilibrium phase change process is accurately captured by the developed two-phase flow model. Based on the detailed validations using the aforementioned two cases, it can be concluded that the developed condensing flow model is accurate to predict the wall pressure and droplet size related to the condensation behaviour in steam turbines. 

### 3.2. Influence of Superheated Steam on Condensing Flows in Turbine Blades

**Figure 6:**
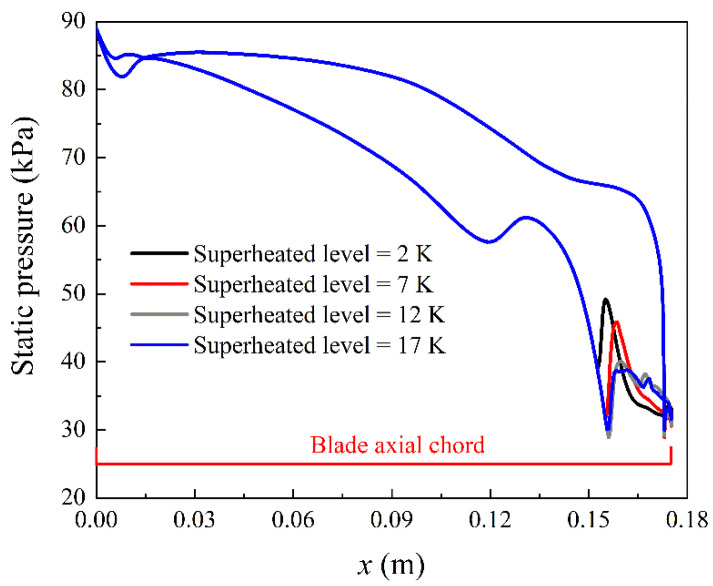
Influence of the inlet superheated level on the pressure distribution in blade cascades.

**Figure 7:**
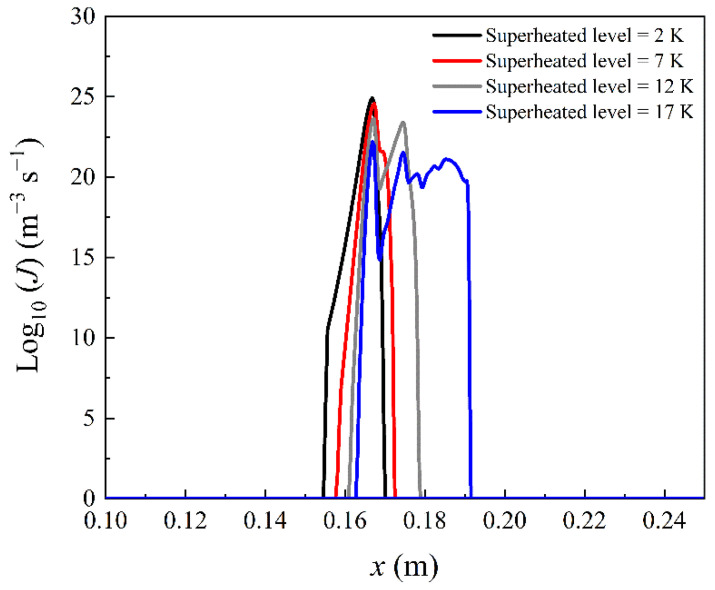
Influence of the inlet superheated level on the nucleation rate along the periodic boundary in the flow channel of linear blade cascades.

**Figure 8:**
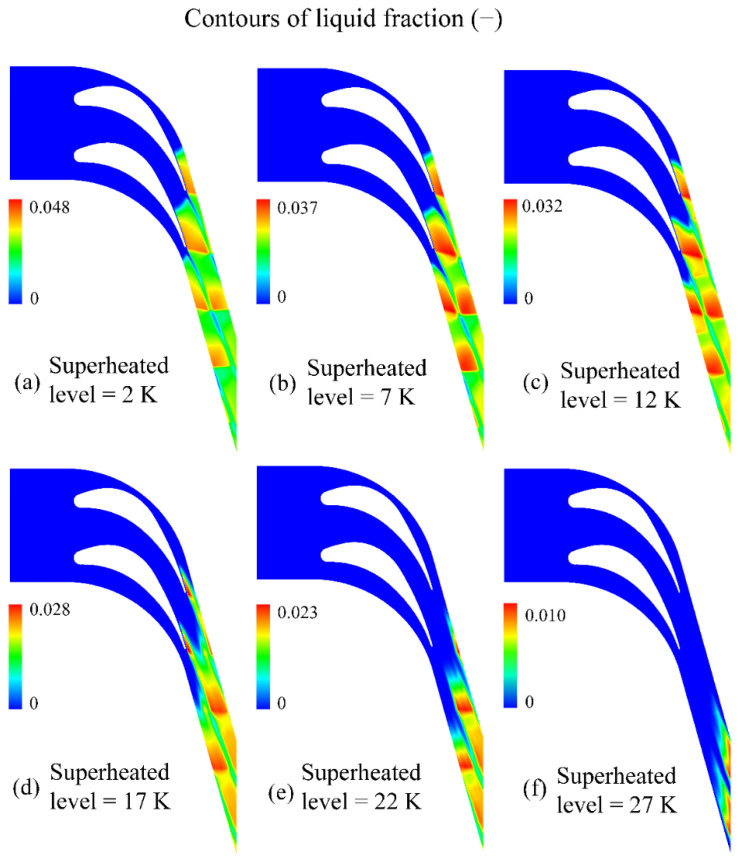
Influence of the inlet superheated level on the liquid fraction in linear blade cascades.

Here we investigate the influence of superheated levels on the nonequilibrium condensation and losses inside blade cascades. The superheated level is defined as the temperature difference between the local vapour temperature and saturation temperature. The inlet superheated levels from 2 to 27 K are evaluated in this section. Figure 6 and Figure 7 only show the effect from 2 to 17 K as this makes it concise as the condensation region moves away from the blade as seen in Figure 8.

Figure 6 describes the blade wall pressure distribution in the linear cascade at the inlet superheated level of 2, 7, 12 and 17 K. Figure 7 illustrates the nucleation rate along the periodic boundary in the flow channel. It can be observed that the Wilson point moves downstream with the increase of the inlet superheated level of the steam. This indicates that the Wilson point is delayed by increasing the inlet steam temperature. Furthermore, the condensation shock of the nonequilibrium condensation is weakened by increasing inlet superheated levels. The maximum value of the nucleation rate decreases with the rises of the inlet superheated levels. This implies that the release of the latent heat during phase change processes inside linear blade cascades is dramatically influenced by the inflow steam superheated level for a steam turbine.

Figure 8 illustrates the liquid fraction inside the linear blade cascade at the inlet superheated level of 2, 7, 12, 17, 22 and 27 K, respectively. On the one hand, the liquid fraction distribution is postponed further downstream the flow channel. The liquid mass fraction has moved away from the blade region when the inlet superheated level of the steam increases to 22 K. Specifically, the liquid fraction is restrained to the near-exit region inside linear blade cascades with an inlet superheated level of 27 K. On the other hand, the maximum value of the liquid fraction decreases significantly with an increasing inlet superheated level inside linear blade cascades. For instance, the liquid fraction can reach approximately 0.048 with the inlet superheated level of 2 K, while it decreases to 0.010 with an inlet superheated level of 27 K. This indicates that the liquid fraction can be weakened by around 79% by increasing the inlet superheated level of 25 K for the turbine blade.

Equation (9) is suggested to assess the condensation loss in steam turbines [41,42].

(9) $$\eta =\zeta (m_{2}-m_{1})h_{fg}$$

where *η* and *ζ* are condensation loss and model coefficient, *m*_1_ and *m*_2_ are liquid flow rates at the entrance and exit of the blade cascade.

**Figure 9:**
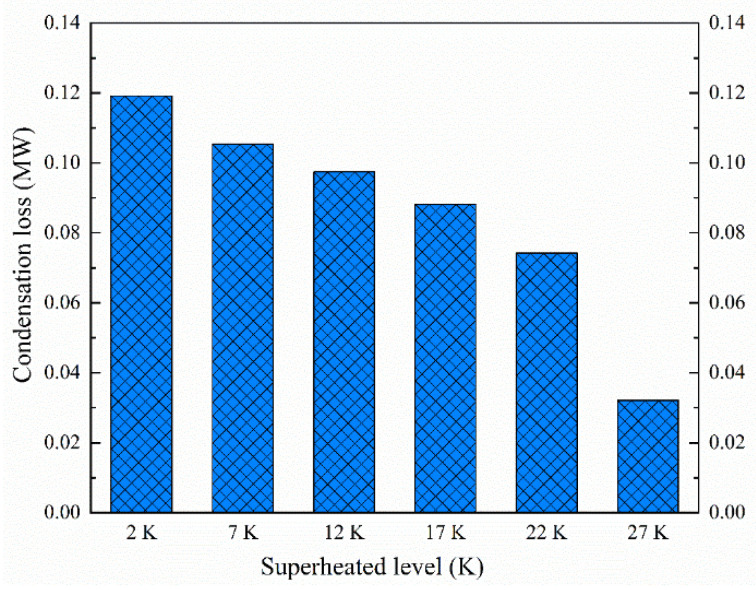
Effect of inlet superheated levels on condensation losses in linear blade cascades.

The influence of the inlet superheated level of the steam on the condensation loss is evaluated based on Equation (9) for the linear blade cascade. Figure 9 depicts the condensation loss at the inlet superheated level of 2, 7, 12, 17, 22 and 27 K, respectively. The increasing inlet superheated level of the steam decreases remarkably with the condensation loss inside blade cascades. The condensation loss is up to 0.12 MW for the inlet superheated level of 2 K, which is weakened to 0.032 MW for the inlet superheated level of 27 K. It indicates that an increase in the inlet superheated level of 25 K can reduce the condensation loss by approximately 73% in blade cascades.

### 3.3. Novel Dehumidification Strategy via Turbulent Disturbance

**Figure 10:**
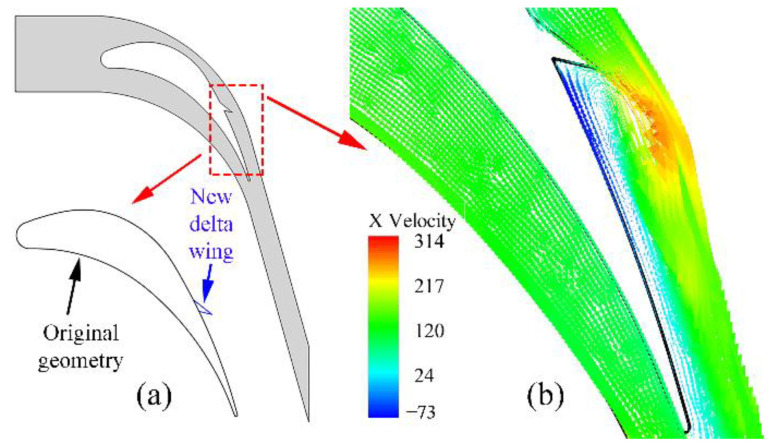
Novel dehumidification strategies via turbulent disturbance in turbine blades: (**a**) new geometry and (**b**) velocity vectors.

In this section, we propose a novel dehumidification strategy for the turbine blades, which installs a delta wing on the suction side of the blade, as shown in Figure 10. The idea is expected to generate strong turbulent disturbances in the transonic flows and subsequently alters the flow behaviour and phase change phenomenon in the condensing flows. It can be seen from the velocity vectors in Figure 10b that the delta wing disturbs the flow field in the turbine blades; in particular, this insert induces the strong vortex downstream of the delta wing that significantly changes the velocity magnitude and directions. The vortex influences the entire flow structures downstream of the delta wing on suction sides, which will influence condensation characteristics within the transonic flow inside the turbine blades.

**Figure 11:**
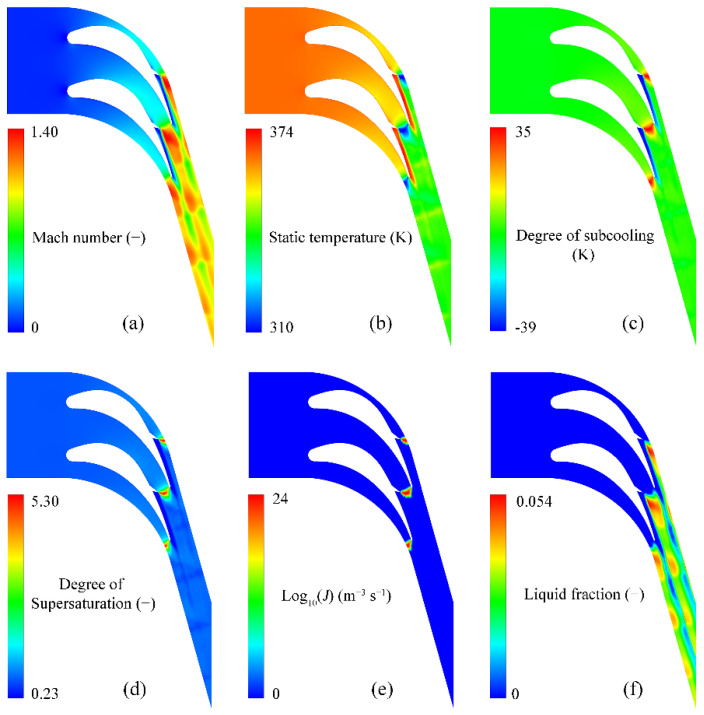
Flow behaviour in the newly designed turbine blades: (**a**) Mach number; (**b**) static temperature; (**c**) degree of subcooling; (**d**) degree of supersaturation; (**e**) nucleation rate and (**f**) liquid fraction.

Figure 11 illustrates the flow behaviour of the steam in the newly designed turbine blades including the Mach number, static temperature, degree of subcooling, degree of supersaturation, nucleation rate and liquid fraction. The turbulent disturbances due to the delta wing dramatically break the transonic flows downstream the wing. The near-wall region presents the subsonic flow, where the Mach number is less than unity. It can be seen from the distribution of Mach number in Figure 11a that there is a large region of subsonic flow along the suction blade wall downstream the delta wing, which subsequently induces the increase in the static temperature in this region that can be defined as the “delta wing affecting region”. It, therefore, results in the superheated steam with a degree of subcooling of about −37 K. The degree of supersaturation lowers to 0.27 downstream of the delta wing, which demonstrates that the steam is far from the nonequilibrium state that cannot induce the steam condensation. 

**Figure 12:**
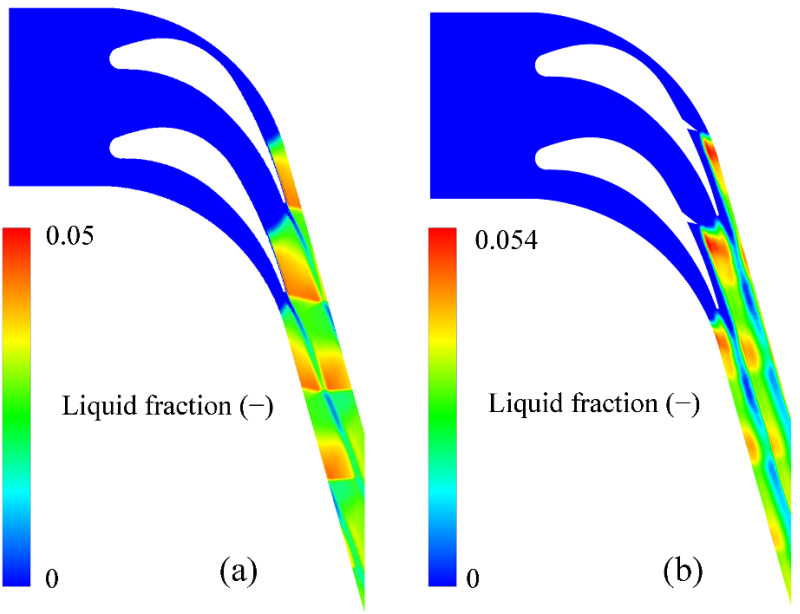
Comparison of liquid fractions between the original geometry (**a**) and new design (**b**) of the linear turbine blade.

Apart from the “delta wing affecting region”, the existence of the delta wing increases the maximum Mach number to 1.40 compared to 1.29 for the original geometry of the turbine blade. This leads to a maximum degree of subcooling of approximately 35 K and the peak degree of supersaturation of around 5.30, which demonstrates that the steam reaches an extremely nonequilibrium state to generate spontaneous condensations within a transonic flow. In these newly designed turbine blades, the nucleation rates are about 10^24^ m^−3^ s^−1^ to generate strong homogeneous nucleation. The nucleation region for these newly designed turbine blades stays at the top of the delta wing, where the maximum degree of subcooling and maximum degree of supersaturation are generated due to the high expansion of the steam. Figure 12 compares the detailed liquid fractions generated in the original and newly designed blades. The liquid phase is far from the blade wall on the suction side although the maximum liquid fraction can reach 0.054 of the total mass, which is approximately 8% higher than the one for the original geometry.

**Figure 13:**
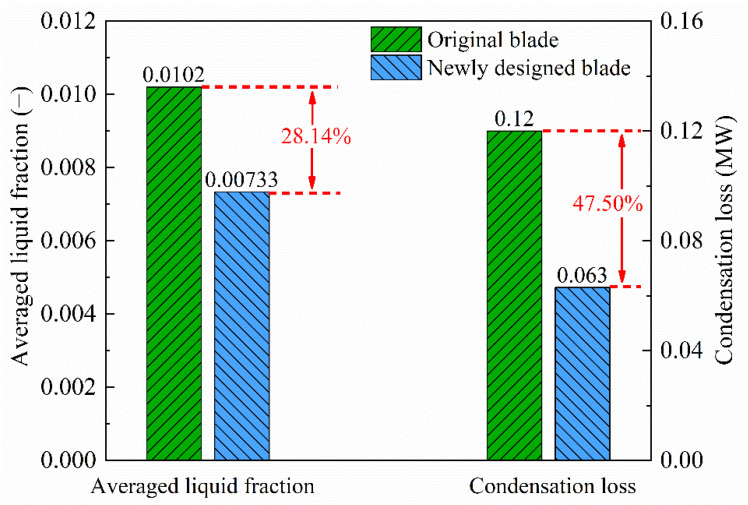
Averaged liquid fraction and condensation loss in the original and newly designed turbine blades.

The comparison of averaged liquid fraction and condensation loss between the original turbine blades and newly designed geometry is shown in Figure 13. It can be seen that the novel dehumidification strategy via installing a delta wing on the blade wall on the suction side can significantly reduce 28.14% averaged liquid fraction, which decreases to 0.00733 for the newly designed blade compared to 0.012 for the original geometry. Furthermore, the condensation loss is only about 0.063 MW for the newly designed geometry compared to 0.12 MW for the original blades. This indicates that the novel design can dramatically reduce the condensation loss by 47.50%, which may improve the energy efficiency in the newly designed turbine blades.

However, it can be observed that the flow separation caused by the delta wing could have a negative impact on the blade performance, such as the aerodynamic efficiency of the turbine stage and the kinetic energy loss. The total loss is evaluated via the following formula suggested by Kermani and Gerber [43], which considered the effect of the aerodynamic and condensation losses in a steam turbine.

χ = (*S_c_* − *S_in_*)/*C_v_* (10)

where χ is the total loss, and *S_c_* and *S_in_* are the entropy at the calculation point and inlet, respectively. *C_v_* is the specific heat in a constant volume.

**Figure 14:**
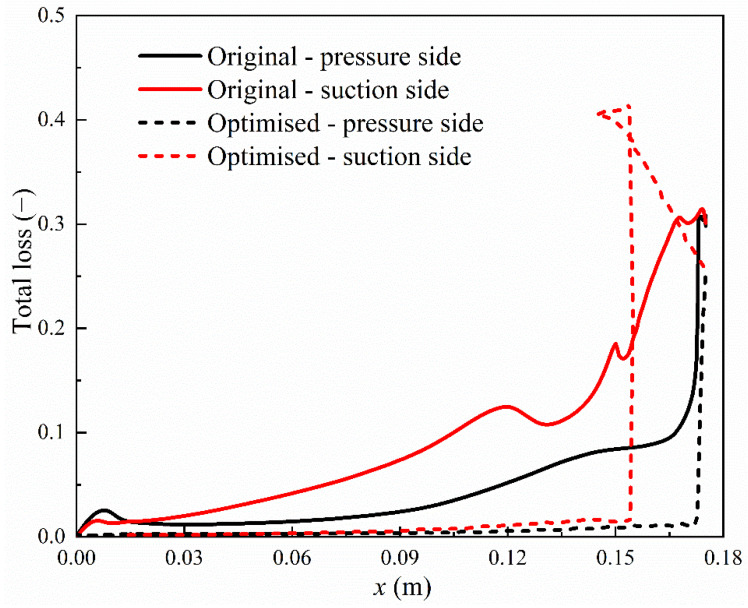
Total losses at the pressure side and suction side of the original and optimised blades in steam turbines.

**Figure 15:**
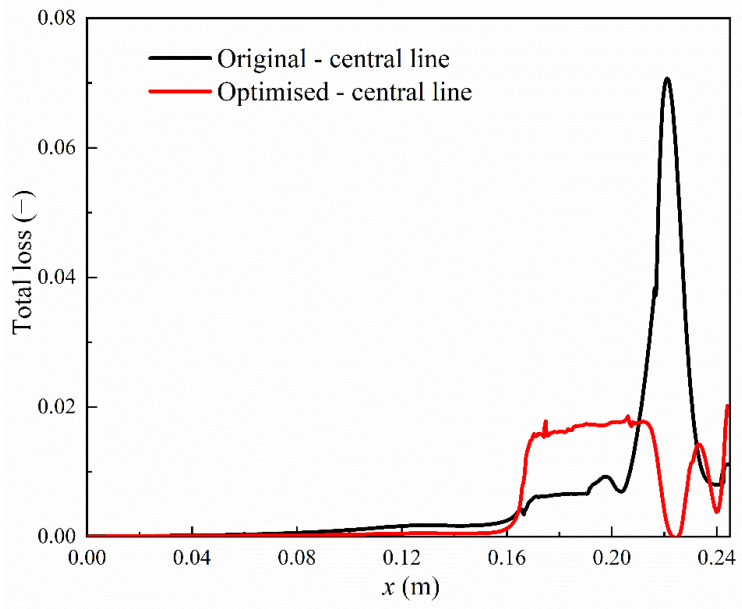
Total losses at the central line of the original and optimised blades in steam turbines.

The total losses on the blade walls on the pressure side and suction side are compared for the original geometry and optimised blades, as shown in Figure 14. In the upstream region of the steam condensation, the total losses in the optimised blade are much smaller than the original geometry both on the pressure side and suction side. However, in the optimised blade, the delta wing induces the flow separation in the downstream region of the condensation on the suction side, which results in a steep increase in the total loss. The maximum value of the total loss is approximately 0.41 for the optimised blade while the original geometry produces around a maximum total loss of 0.32. This indicates that the total loss increases by 28% by the optimised blades compared to the original one. The total losses are also compared in the flow channel in the original and optimised blades, i.e., at the central line as shown in Figure 15. It can be seen that the optimised blade produces higher total losses in the downstream region of the condensation (0.16 m < *x* < 0.18 m) compared to the original geometry. 

In general, the novel dehumidification strategy via installing a delta wing on the blade wall on the suction side can generate intensively turbulent disturbances in the flow field, which not only reduces the averaged liquid fraction due to the nonequilibrium condensation but also keeps the liquid phase away from the blade wall. This reduces the influences of the generation of liquid phases on the blades and enables high reliabilities of the turbines. Meanwhile, the condensation loss is significantly reduced in the newly designed turbine blades which improve energy efficiencies. However, the optimised blade also results in higher total losses by 28% compared to the original geometry. It is also necessary to consider the effect of the added delta wing on the blade structure, the stresses during operation and the ease of manufacturing. These issues need to be further investigated in future studies, including the structure of the delta wing, the installation position of the added wing as well as the delta wing angle. Nevertheless, the novel strategy could provide a view for improving the dehumidification performance of turbine blades.

## 4. Conclusions

The wet steam two-phase flow model was developed to assess the phase change behaviour in a linear blade cascade in steam turbines. We proposed a new concept to improve the dehumidification performance based on the turbulent disturbance on the blade suction side. The effect of the inlet operating parameter on the wet steam two-phase flow in the linear blade cascade is evaluated in detail. The increase in the inlet superheated level of the steam delayed the onset of the nonequilibrium condensation and decreased the nucleation rate, liquid fraction and condensation loss. The increase of the inlet superheated level of 25 K can reduce 79% liquid fraction and 73% condensation loss in linear blade cascades, respectively. In newly designed turbine blades, the averaged liquid fractions and condensation losses were significantly reduced by 28.1 and 47.5% compared to the original geometry. Meanwhile, the delta wing installing on the blade wall on the suction side dramatically kept the liquid phase region away from the walls of the blade to reduce the interaction of the liquid phase and blades.

## Figures and Tables

**Table 1:** The dimensions of the linear blade cascade [39].

Pressure Side				Suction Side			
*x* (mm)	*y* (mm)	*x* (mm)	*y* (mm)	*x* (mm)	*y* (mm)	*x* (mm)	*y* (mm)
0	143.45	126.24	79.17	0	143.45	137.49	97.34
14.1	132.95	135.57	68.23	14.47	156.15	144.97	82.50
28.3	132.30	140.01	62.51	30.55	160.71	148.52	74.95
42.38	130.19	144.22	56.70	47.36	163.46	151.91	67.40
56.16	126.65	148.25	50.76	64.59	162.75	155.19	59.74
69.52	121.72	152.05	44.76	81.21	158.12	158.29	52.10
82.28	115.45	155.66	38.65	96.36	150.05	161.28	44.37
94.35	107.91	159.08	32.43	109.68	139.29	164.1	36.66
105.63	99.14	162.36	26.00	120.74	126.24	166.8	28.85
116.23	89.50	165.45	19.41	129.5	111.89	169.36	20.99

**Table 2:** Boundary conditions for linear blade cascades.

Boundary Conditions	Blade Inlet	Blade Outlet	Fluid and Walls
Total pressure	89,000 Pa	39,000 Pa	Wet steam flow No-slip, adiabatic walls

## Data Availability

The research data supporting this publication are provided within this paper.
